# Survey of a nutrition management method for very low birthweight infants: Status before wide use of breast milk banks in Japan

**DOI:** 10.1111/ped.14074

**Published:** 2020-02-27

**Authors:** Kosuke Oikawa, Motoichiro Sakurai, Tetsuro Murakawa, Reita Kidokoro, Yuya Nakano, Hideyuki Asai, Hirotaka Ochiai, Takako Shirasawa, Takahiko Yoshimoto, Akira Minoura, Akatsuki Kokaze, Katsumi Mizuno

**Affiliations:** ^1^ Department of Pediatrics Showa University School of Medicine Tokyo Japan; ^2^ Department of Hygiene, Public Health and Preventive Medicine Showa University School of Medicine Tokyo Japan

**Keywords:** donor milk, neonatologists, nutrition, questionnaire survey, very low birthweight infant

## Abstract

**Background:**

The importance of breast‐feeding for very low birthweight (VLBW) infants has been pointed out. Some overseas studies suggested that the standardization of enteral nutrition (EN) leads to improved prognosis in VLBW infants. In Japan, however, physicians in charge of infants are responsible for making nutrition management decisions on an individual basis. We conducted an online survey to clarify the course of nutrition management of VLBW infants currently implemented in Japan.

**Methods:**

We mailed a notice to 300 representative neonatologists throughout Japan requesting their participation in the online survey. On the survey website, neonatologists responded to questions regarding the nutritional strategy for five birthweight groups (less than 500 g, 500–749 g, 750–999 g, 1,000–1,249 g and 1,250–1,499 g).

**Results:**

Responses were recieved from 137 neonatologists. The first choice for EN up to 1 week after birth was breast milk regardless of birthweight (92.0% for 1,250–1,499 g to 95.6% for 500–999 g). More than 30% of the respondents answered that they fast infants who weigh <750 g at birth or feed them with other mothers’ breast milk until their own mother’s milk becomes available. The lower the birthweight, the later EN is started, and the greater the number of days to establish EN.

**Conclusion:**

The lower the birthweight, the more difficult it is to feed infants their own mother’s milk and the later the EN is started. If donor milk is supplied in a stable manner, it takes fewer days to establish EN.

Recently, the survival rate of very low birthweight (VLBW) infants has improved due to advances in the medical care of preterm infants.^1^ Many facilities have introduced new nutritional strategies for VLBW infants, including early aggressive nutrition (EAN)^2^, in which parenteral nutrition is started as early as the day of birth and feeding with milk is started early. This trend reflects growing recognition of the necessity of starting to supply nutrition as early as possible after birth, based on the idea that, in preterm births, infants fall into nutritional emergency as the nutritional supply from the placenta stops immediately after birth. For very premature infants, in particular, it is considered important to recommend feeding them with breast milk (BM) to reduce the risk of diseases including necrotizing enterocolitis (NEC).^3^ The mortality rate of VLBW infants and very preterm infants, and the morbidity of NEC are lower in Japan than in Europe and the USA. The reason for this is thought to be due to differences in nutrition management methods. However, there are no universal guidelines for nutrition management for VLBW infants in Japan. We speculated that physicians in charge of infants at individual facilities make decisions on the course of nutrition management. In recent years, milk banks in Japan have started to supply donor,^4^ which has made it possible to standardize nutrition management using human BM early after birth. Nutritional management methods in preterm infants are expected to change in Japan. We therefore conducted a nationwide online survey of neonatologists to clarify the course of nutrition management for VLBW infants.

## Methods

We mailed a notice about the survey to all 300 representative neonatologists certified by the Japan Society of Perinatal and Neonatal Medicine throughout Japan and we also asked their co‐workers to participate in this survey. We also used the website of the Japanese Human Milk Bank Association (JHMBA) to ask neonatologists to participate in a survey regarding the nutritional strategy for five birthweight groups. The survey period was from December 25, 2017, to January 31, 2018. In the survey questionnaire, the birthweight of infants was categorized into the following five groups: less than 500 g, 500–749 g, 750–999 g, 1,000–1,249 g and 1,250–1,499 g (Appendix 1). This survey was approved by the ethical committee of Showa University Koto Toyosu Hospital (Approval No. 17T5021) and research information was published on the website.

## Results

### Background of respondents and their facilities

A total of 137 neonatologists responded. The number of years of experience in neonatal care was <5 years for four respondents, 5–9 years for 21 respondents, 10–19 years for 69 respondents, 20–29 years for 33 respondents and ≥30 years for 10 respondents. The number of VLBW infants they take care of per year was <5 for 49 respondents, 5–9 for 40 respondents, 10–19 for 38 respondents, 20–29 for seven respondents and ≥30 for three respondents.

### Survey questions and results

Questions are categorized in the items “protocol for nutrition management,” “general practice for enteral nutrition (EN),” “enteral feeding in specific situations,” “gastric residual content,” “parental nutrition,” and “others.” Several questions are presented in  Appendices 1, 2, 3 and 1, 2, 3.

#### Protocol for nutrition management

Question 1: “Is there any standard course of nutrition management (written) of VLBW infants at your facility?” Forty‐eight respondents (35%) answered that there is a standard course; 87 (63%) answered that there is no standard course and two did not answer.

#### General practice for enteral nutrition (EN)

Question 2: “When, approximately, do you start enteral nutrition (EN) (injection)? (by weight group).”

**Figure 1:**
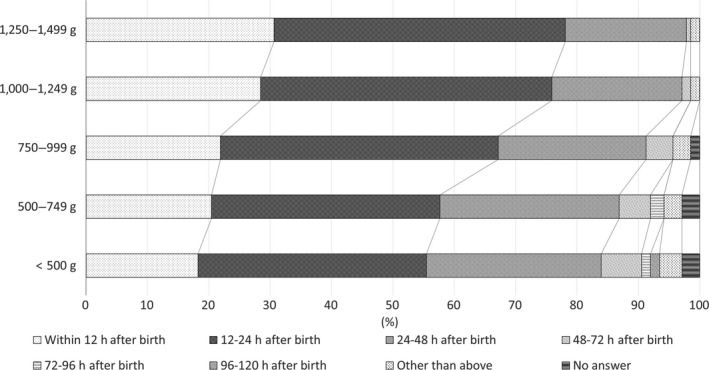
When, approximately, do you start EN (injection)? (*n* = 137).

The largest number of respondents answered that they start 12–24 h after birth for all weight groups, followed by 24–48 h after birth for the three groups with birthweight ≤999 g, and within 12 h after birth for the two groups with birthweight ≥1,000 g (Fig. 1).

Question 3: “What do you mainly choose for EN up to 1 week after birth? (by weight group).”For all weight groups, more than 90% (92–96%) of the respondents answered that they choose BM, followed by milk for low birthweight infants (1–4%), and other (1–2%).

Question 4: “What do you do when the mother’s BM cannot be administered? (by weight group).”

**Figure 2:**
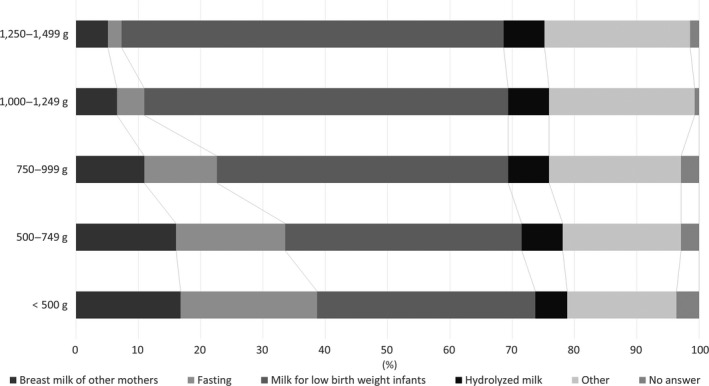
What do you do when the mother’s BM cannot be administered? (*n *= 137).

The lower the birthweight, the lower the percentage of respondents that answered that they use milk for low birthweight infants and the higher the percentage that answered that they choose fasting. As birthweight decreased, the respondents answered more often that they use other mothers’ milk. For the two groups with birthweight up to 749 g, approximately 16% of the respondents answered that they use other mothers’ milk (Fig. 2).

Question 5: “How many times a day do you give EN at the start? (by weight group).” In all groups, the largest number of respondents answered that they give EN eight times (55% <500 g, 69% for 500–749 g, 82% for 750–999 g, 91% for 1,000–1,249 g and 94% for 1,250–1,499 g). The lower the birthweight, the greater the number of respondents that answered that they give EN (injection) four times (25% for < 500 g, 16% for 500‐749 g, 6% for 750–999 g, 1% for 1,000–1,249 g and 1% for 1,250–1,499 g).

**Figure 3:**
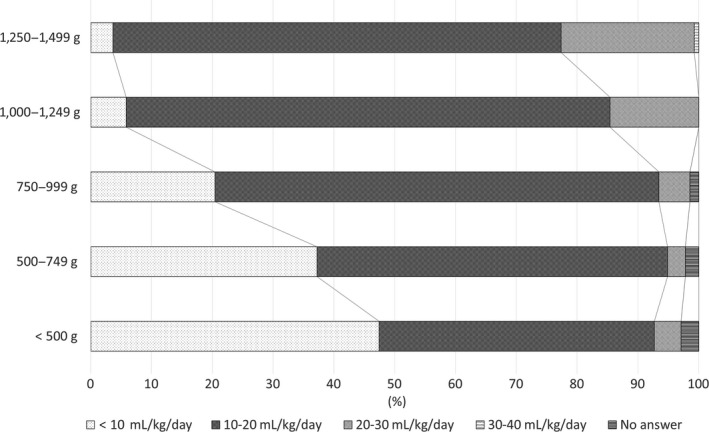
How much do you increase the volume of EN per day? (*n* = 137).

Question 6: “How much do you increase the volume of EN per day? (by weight group).” For the groups with birthweight <500 g, the number of respondents who answered that they start at a rate of <10 mL/kg/day was almost the same as the number who answered that they start at 10–20 mL/kg/day. As the birthweight increased, more respondents answered that they start at 10–20 mL/kg/day (Fig. 3).

**Figure 4:**
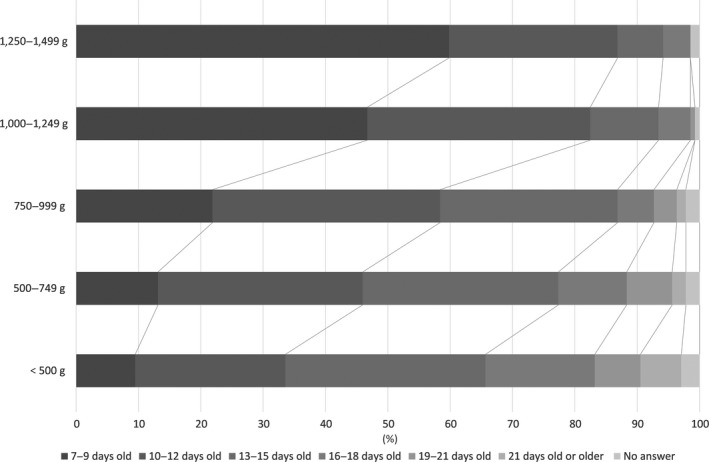
At which age in days, approximately, is EN established (volume reaches 100 mL/kg/day)? (*n* = 137).

Question 7: “At which age in days, approximately, is EN established (volume reaches 100 mL/kg/day)? (by weight group).” For birthweights <500 g, the largest number of respondents (32%) answered 13–15 days old, followed by 10–12 days old (24%) and 16–18 days old (18%). For 500–749 g and 750–999 g, the largest number of respondents answered 10–12 days old (33% for 500–749 g and 36% for 750–999 g), followed by 13–15 days old (31% for 500–749 g and 28% for 750–999 g). For birthweights 1,000–1,249 g and 1,250–1,499 g, the largest number of respondents answered 7–9 days old (47% for 1,000–1,249 g and 60% for 1,250–1,499 g), followed by 10–12 days old (36% for 1,000–1,249 g and 27% 1,250‐1,499 g) (Fig. 4).

#### Enteral feeding in specific situations (sedation, indomethacin administration, transfusion, extubation)

Question 8: “How do you proceed with EN during sedation (number of dosing)? (by weight group).”

For each group, the largest number of respondents answered eight times (38% for <500 g, 47% for 500–749 g, 53% for 750–999 g, 57% for 1,000–1,249 g and 58% for 1,250–1,499 g). The lower the birthweight, the greater the number of respondents that answered four times (14% for <500 g, 8% for 500–749 g, 1% for 750–999 g, 1% for 1,000–1,249 g and 1% for 1,250–1,499 g).

Question 9: “How do you proceed with EN during sedation (rate of increase in volume)? (by weight group).” For birthweights <500 g and 500–749 g, the largest number of respondents answered 10 mL/kg/day (38% for <500 g and 33% for 500–749 g), followed by 10–20 mL/kg/day (25% for <500 g and 31% for 500–749 g). For 750–999 g, 1,000–1,249 g and 1,250–1,499 g, the largest number of respondents answered 10–20 mL/kg/day (37% for 750–999 g, 44% for 1,000–1,249 g and 43% for 1,250–1,499 g), followed by 10 mL/kg/day (26% for 750–999 g, 12% for 1,000–1,249 g and 12% for 1,250–1,499 g).

Question 10: “Do you change the course of EN management during the administration of indomethacin?” Eighty‐one respondents (59%) answered that they do not change the course and 39 (28%) answered that they reduce the dose. The answers of 16 respondents were other than the above, including that, depending on the case, they make decisions based on radiographs, hemodynamics, or blood flow in the superior mesenteric artery. One respondent did not answer.

Question 11: “Do you change the course of EN management during transfusion?” One hundred thirty respondents (94%) answered that they do not change the course, five answered that they reduce the dose, one answered that he or she stops feeding and the answer of the remaining respondent was other than the above.

Question 12: “Do you set a fasting period before or after extubation? How long is the fasting period if you set it?” Seventy‐four respondents (54%), accounting for approximately half of the respondents, answered that they set a fasting period before extubation. For the duration, the largest number of respondents, 45 (60%), answered 3–4 h, followed by 23 respondents (31%) answering 1–2 h. After extubation, approximately half of the respondents, 65 (47%), answered that they set a fasting period. The largest number of respondents, 23 (35%), answered that the duration is 3–4 h, followed by 18 respondents (27%) who answered 1–2 h.

Question 13: “Do you use probiotics? When do you start?” Ninety‐nine respondents (72%) answered that they use probiotics. Almost the same number of respondents (47 and 46 respondents) answered that they start at 0 days of age or that they start at 1 day of age.

#### Gastric residual content

Question 14: “Tell us about the response to residual gastric content (undigested substance without bile or blood) before feeding (assuming there is no problem in systemic condition and abdominal findings). (free description response).”

Fifty‐three percent of the respondents answered that they implement a certain intervention for 50–60% of gastric content remaining, followed by 19% who answered that they implement a certain intervention for 30–40% of gastric content remaining. For 50–60% of gastric content remaining, 48% of the respondents answered that they stop feeding and 38% answered that they skip feeding (once). For 30–40% of gastric content remaining, 40% of the respondents answered that they stop feeding and 32% answered that they planned injection volume minus the volume of gastric content. For 10–20% of gastric content remaining, 75% of the respondents answered that they give the difference in the amount. For 70% or more of gastric content remaining, many respondents answered that they stop feeding.

Question 15: “Tell us about the discontinuation (interruption) criteria for EN. (free description response).”

Most respondents answered that they discontinue in cases of thick bile or bloody aspirate. Other answers included that they discontinue EN when infants vomit more than once, when more than half of the previous feeding remains in stomach for two consecutive times, and at the discretion of the physician in charge of the infant.

#### Parenteral nutrition

Question 16: “What volume of EN do you generally need to discontinue parenteral nutrition? (by weight group).” For each group, the largest number of respondents answered 100 mL/kg/day (43% for <500 g 44% for 500–749 g, 47% for 750–999 g, 54% for 1,000–1,249 g and 54% for 1,250–1,499 g), followed by 120 mL/kg/day (35% for <500, 36% for 500–749 g, 34% for 750–999 g, 30% for 1,000–1,249 g and 30% for 1,250–1,499 g).

Question 17: “Do you perform parenteral nutrition early after birth? If yes, when do you start amino acids after birth? (by weight group).”Except for one respondent, all answered that they perform parenteral nutrition early after birth. All the weight groups showed a similar tendency: 34% of the respondents answered that they start upon hospitalization, 32–36% answered that they start within 24 h after birth, and 26–27% answered that they start between 24 and 72 h after birth.

Question 18: “What is the starting dose and the target maximum dose of amino acids for intravenous administration (by weight group).”

For <500 g, 500–749 g and 750–999 g, many respondents answered that they start at 2.0 g/kg/day (29% for <500 g, 47% for 500–749 g and 29% for 750–999 g), followed by 3.0 g/kg/day (27% for <500 g, 20% for 500–749 g and 27% for 750–999 g) and 2.5 g/kg/day (21% for <500 g, 11% for 500–749 g and 22% for 750–999 g). For 1,000–1,249 g and 1,250–1,499 g, many respondents answered that they start at 3.0 g/kg/day (29% for 1,000–1,249 g and 30% for 1,250–1,499 g), followed by 2.0 g/kg/day (27% for 1,000–1,249 g and 28% for 1,250–1,499 g) and 2.5 g/kg/day (23% for 1,000–1,249 g and 22% for 1,250–1,499 g). For <500 g, 500–749 g and 750–999 g, many respondents answered that they set the target maximum dose to 2.0 g/kg/day (29% for <500 g, 47% for 500–749 g and 29% for 750–999 g). For 1,000–1,249 g and 1,250–1,499 g, many respondents answered that they set it to 3.0 g/kg/day (29% for 1,000–1,249 g and 30% for 1,250–1,499 g).

Question 19: “Roughly, when you start and stop the use of fat emulsion?” Most respondents answered that they start at the age of 1–3 days considering respiration, jaundice and infection control. Regarding the end of use, the proportion of respondents who answered that they stop when the volume of EN reaches around 100–120 mL/kg/day and that they stop at the completion of parenteral fluid, combined, was 80%. Six percent of the respondents answered that they do not use it, in principle.

Question 20: “At which dose in g/kg/day do you start the administration of fat emulsion and at which dose in g/kg/day do you stop the dose increase?” One hundred and three respondents (75%) answered that they start at 0.5 g/kg/day and 21 respondents (15%) answered that they start at 1 g/kg/day. The target was mentioned as 2 g/kg/day by the largest number of respondents (56%), followed by 3 g/kg/day (11%).

#### Others

Question 21: “What percentage of the parenteral nutrition do you think is the amount of water absorbed by EN?” Thirty‐nine respondents (28%) answered that the water content of EN is 80% compared to the water content of parenteral nutrition and 39 (28%) answered that it is 100%.

Question 22: “When you restrict fluid in infants with chronic lung disease or patent ductus arteriosus, to what extent do you restrict?” The largest number of respondents, 51 (37%), answered that they restrict to 120 mL/kg/day, followed by 23 respondents (16%) answering that they restrict to 100 mL/kg/day.

## Discussion

Nutrition management of VLBW infants has changed greatly. Additive powders to enhance the nutrition that is lacking in BM were developed, and then, the concept of probiotics was established. Recently, very early EN and EAN have been introduced. In Europe and the USA, EAN is recommended for nutrition management for low birthweight infants, and BM banks are available in some areas. The JHMBA was founded in 2017 in Japan. The future establishment of local BM banks and the anticipated use of donor milk (DM) at neonatal intensive care units (NICUs) as a standard treatment would affect the course of nutrition management of VLBW infants in Japan.^4^ We surveyed the status of nutrition management of VLBW infants before the widespread use of DM.

The facilities of more than half of the respondents did not have any standard course of nutrition management of VLBW infants, suggesting that the management of VLBW infants depends on the facilities where infants were born or the physicians in charge of them. Our survey indicated that the lower the birthweight, the later EN is started, the smaller the amount of nutrition per day, and the higher the number of days to establish EN.

Many respondents answered that they start EN roughly 12–24 h after birth for all weight groups. However, our survey showed that the lower the birthweight, the later the start of EN, which was probably because the systemic condition of the infants and the secretion of BM were considered in determining when to start it. Hamilton *et al*. reported that EN starting 14 h after birth in VLBW infants shortened the period of parenteral nutrition and did not increase the onset of NEC. In the USA and Europe, early EN in cases of good systemic condition of infants is being established as a standard treatment.^5^

To start EN around 12 h after birth in extremely premature infants, the mother’s BM needs to be obtained early. However, this may be difficult. In our survey, most respondents answered that they select BM for nutrition up to 1 week after birth and that, when the mother’s BM is insufficient, they most frequently use artificial milk for low birthweight infants. Nevertheless, for infants with lower birthweight, the respondents were shown to avoid the use of artificial milk. As a result, the fasting period is prolonged or other mothers’ BM is used. As the fasting period is prolonged, feeding intolerance is likely to occur and it would take more time to establish EN.^6^, ^7^ Moreover, other mothers’ milk is associated with the risk of viral or bacterial infection when it is unpasteurized. The odds ratio of being exposed to pathogens was reported to be 1.31 when infants are fed with other mothers’ milk for 1 month.^8^ A basic study in newborn pigs showed that 40% of all calories need to be taken through the digestive tract to maintain the growth of the digestive tract.^9^ Continuous and safe EN is also desirable for humans. In countries with sufficient BM banks, DM can be used and there are no concerns regarding unnecessary prolongation of the fasting period or onset of infection when the mother’s BM is insufficient. Furthermore, in nutrition management of premature infants early after birth, there is less need for artificial milk and the risk of NEC is considered to be lower if there are sufficient milk banks.^10^ A previous study showed that the use of DM lowered the incidence of viral infection, decreased visits to clinics after discharge from NICU and reduced medical costs.^11^

Many respondents answered that they start to increase the volume of EN by 10–20 mL/kg/day per day. A study comparing a group with a higher daily increase rate of EN (15–20 mL/kg/day) with a group with a lower rate of increase (30–40 mL/kg/day) did not show any significant difference in the onset of NEC between these groups and suggested that a faster increase is more beneficial as full feeding is achieved earlier.^12^, ^13^ The number of days taken to establish EN tended to be greater for infants with lower birthweight. This may be attributed to the environment in which infants cannot ingest BM. If DM is supplied in a stable manner in Japan, the increase in the feed volume would be easily achieved regardless of the facility and the number of days to the establishment of EN would be smaller.

In our study, we asked questions regarding various factors associated with nutrition management. The answers showed that, at many facilities, infants born at less than 26 weeks’ gestation are sedated until 72 h after birth. We did not ask questions about the drugs used for sedation or the depth of sedation. Many respondents answered that they give roughly 10 mL/kg/day in about eight divided doses per day in EN during sedation, suggesting that the adverse effect of fasting and the importance of EN management are recognized.

About 60% of the respondents answered that they do not change nutrition management during the administration of indomethacin sodium. The remaining 40% of the respondents answered that they make certain changes, suggesting that they adjust the course of EN considering the impact of indomethacin sodium on the blood flow in the digestive tract.^14^ Respondents had different opinions regarding nutrition management during the administration. For example, one respondent stated that EN depends on clinical judgment, based on the results of ultrasonography. Continuation of feeding with human milk during the administration of indomethacin was reported not to lead to significant differences in the onset of NEC or the age in days of achievement of full EN.^15^ Further study in Japan is desired.

More than 90% of the respondents answered that blood transfusion does not affect the course of EN. A previous review suggested that fasting during transfusion significantly decreased the incidence of transfusion‐related NEC,^16^ while not transfusion, but severe anemia was reported to be associated with the increase in the risk of NEC in VLBW infants.^17^ Although no consistent conclusions have been reached, our survey showed that fasting is avoided in many facilities.

Open‐ended questions were asked regarding how to deal with residual gastric content and discontinuation criteria for EN. The answers indicated differences in the amount injected in cases of about 10–20% of residual gastric content and infants are fasted in cases of 50–60% or more of residual gastric content in many facilities. No major difference in the measures against residual content between facilities was speculated in clinical practice. As frequent confirmation of residual gastric content was reported to increase the risk of NEC and prolong the time to full feeding^18^ and adjustment of injection volume based not on residual gastric content, but rather measurement of abdominal circumference was reported to be useful for early achievement of full feeding,^19^ standardization of the evaluation method of residual gastric content would be an issue to be solved in the future.

## Limitations

The reliability of the present results is limited because they were based on an online survey and respondents answered anonymously. Recruitment of respondents may possibly have been biased toward facilities highly interested in nutrition methods as participation in the survey was requested through perinatal care facilities throughout Japan or via the Internet. A total of 137 respondents were from 119 facilities overall, and 32 were from 13 facilities. Our survey suggested that the timing for starting EN and the number of days to establishment of EN vary by the physician in charge within the same facility, even within the same NICU where staff members are considered to be highly interested in neonatal nutrition. If the course of nutrition management is determined by each facility, the results of this survey may possibly be influenced by the particular facilities to which relatively more respondents belong. In our survey, the differences in management methods within a facility could not be examined because of the small number of facilities with more than one respondent.

## Conclusion

This study showed that more than 40% of neonatologists fast infants with a birthweight <750 g or feed them with other mother’s BM until their own mother can breast‐feed. The JHMBA was founded in 2017 in Japan and shipment of DM to facilities throughout Japan was started. Currently, DM is requested frequently for VLBW infants or extremely premature infants with complications such as heart or gastrointestinal diseases for whom the professionals in charge hesitate to use artificial milk. If experience in using a milk bank is accumulated in Japan, the use of DM may become a standard nutrition management method for VLBW infants, to be applied early after birth. We conducted this survey to investigate the status before the prevalent use of milk banks. We will continue to investigate future changes in nutrition management.

## Disclosure

The authors declare no conflict of interest.

## Author contributions

K.O., M.S., and K.M. planned the study; H.O., T.S., T.Y., A.M. and A.K. designed the study and examined the statistical validity; and K.O., T.M., R.K., Y.N. and H.A. were in charge of manuscript writing. All authors read and approved the final manuscript.

## Appendix 1 Survey questions

**Table:**

Question 1.	Is there any standard course of nutrition management (written) of VLBW infants at your facility?
	a) Yes b) No c) Unknown
Question 2.	When, approximately, do you start EN (injection)? (by weight group)
	a) Within 12 h b) 12–24 h after birth c) 24–48 h after birth d) 48–72 h after birth
	e) 72–96 h after birth f) 96–120 h after birth g) 120–144 h after birth h) Other
Question 3.	What do you mainly choose for EN up to 1 week after birth? (by weight group)
	a) BM b) Milk for low birthweight infants c) Other
Question 4.	What do you do when the mother’s BM cannot be administered? (by weight group)
	a) BM of other mothers b) Milk for low birthweight infants c) Hydrolyzed milk d) Fasting e) Other
Question 5.	How many times a day do you give EN at the start? (by weight group)
	a) 1–12 times or more b) Continuous feeding
Question 6.	How much do you increase the volume of EN per day? (by weight group)
	a) Less than 10 mL/kg/day b) 10–20 mL/kg/day c) 20–30 mL/kg/day d) 30–40 mL/kg/day e) Over 40 mL/kg/day
Question 7.	At which age in days, approximately, is EN established (volume reaches 100 mL/kg/day)? (by weight group)
	a) 7–9 days b) 10–12 days c) 13–15 days d) 16–18 days e) 19–21 days f) After 21 days
Question 8.	How do you proceed with EN during sedation (number of dosing)? (by weight group)
	a) 1–12 times or more b) Continuous feeding
Question 9.	How do you proceed with EN during sedation (rate of increase in volume)? (by weight group)
	a) Less than 10 mL/kg/day b) 10–20 mL/kg/day c) 20–30 mL/kg/day d) 30–40 mL/kg/day e) Over 40 mL/kg/day
Question 10.	Do you change the course of EN management during the administration of indomethacin?
	a) Fasting b) Reducing the volume c) No change d) Free description response
Question 11.	Do you change the course of EN management during transfusion?
	a) Fasting b) Reducing the volume c) No change d) Free description response
Question 12.	Do you set a fasting period before or after extubation? How long is the fasting period if you set it?
	a) Yes b) No
	If yes, select a) 1–2 h, b) 3–4 h, c) 5‐6 h, d) 7–8 h, e) 9–10 h, f) 11–12 h, g) 12–23 h, or h) More than 24 h
Question 13.	Do you use probiotics? When do you start?
	a) Yes b) No If yes, select a) 0 days, b) 1 day, c) 2 days, d) 3 days, e) 4 days, f) 5 days, g) 6 days
Question 14.	Tell us about the response to residual gastric content (undigested substance without bile or blood) before feeding
	before feeding (assuming there is no problem in systemic condition and abdominal findings)
	(free description response)
Question 15.	Tell us about the discontinuation (interruption) criteria for EN.
	(free description response)
Question 16.	What volume of EN do you generally need to discontinue the parenteral nutrition? (by weight group)
	a) 100 mL/kg/day b) 110 mL/kg/day c) 120 mL/kg/day d) 130 mL/kg/day
	e) More than 130 mL/kg/day f) Do not do parenteral nutrition
Question 17.	Do you perform parenteral nutrition early after birth? If yes, when do you start amino acids after birth? (by weight group)
	a) Yes b) No
	a) Start from hospitalization b) Start within 24 h after birth c) Start within 24–72 h after birth
	d) Start after 72 h after birth e) Upon deciding on adaptation f) Do not do parenteral nutrition
Question 18.	What is the starting dose and the target maximum dose of amino acid for intravenous administration? (by weight group)
	a) 0.5 g b) 1.0 g c) 1.5 g d) 2.0 g e) 2.5 g f) 3.0 g g) 3.5 g h) 4.0 g i) 4.5 g or above
Question 19.	Roughly, when you start and stop the use of fat emulsion?
	(free description response)
Question 20.	At which dose in g/kg/day do you start the administration of fat emulsion and at which dose in g/kg/day do you stop the dose increase?
	a) 0.5 g b) 1.0 g c) 1.5 g d) 2.0 g e) 2.5 g f) 3.0 g g) 3.5 g h) 4.0 g i) 4.5 g or above
Question 21.	What percentage of the parenteral nutrition do you think is the amount of water absorbed by EN?
	a) 40% b) 50% c) 60% d) 70% e) 80% f) 90% g) 100% h) Other
Question 22.	When you restrict fluid in infants with chronic lung disease or patent ductus arteriosus, to what extent do you restrict?
	a) 50 b) 60 c) 70 d) 80 e) 90 f) 100 g) 110 h) 120 i) 130 j) 140 k) 150 l) 160 m) 170 n) 180 mL/kg/day

EN, enteral nutrition; VLBW, very low birthweight.

## Appendix 2

Question 23: “What is the approximate upper limit of acceptable weight loss rate?” (By weight group.) Approximately 40% (40% for <500 g, 37% for 500–749 g, 39% for 750–999 g, 39% for 1,000–1,249 g and 42% for 1,250–1,499 g) of respondents answered that they accept up to 10% weight loss, regardless of weight group. Approximately 80% or more (80% for <500 g, 80% for 500–749 g, 82% for 750–999 g, 90% for 1,000–1,249 g and 89% for 1,250–1,499 g) answered that they accept up to 15% weight loss.

Question 24: “Which time point do you refer to for the weight to use for calculating the fluid volume to give, before the weight is restored to the level at birth?” Seventy‐nine respondents (57%) answered that they use birthweight, 47 (34%) answered that they weigh the infants every day and use that weight to calculate the amount of fluid, one answered that he or she uses the weight at greatest weight loss, and the answers of 10 respondents were other than the above, including that the frequency of weighing is determined based on whether or not respiratory support is used and that the decision on weighing is made based on the age in days.

Question 25: “When does the volume of EN (injection) reach 20 mL/kg/day?” (By weight group.) For the three groups with birthweight up to 999 g, the largest number of respondents answered that it reaches 20 mL/kg/day 48–72 h after birth (23% for <500 g, 26% for 500–749 g and 30% for 750–999 g), followed by 72–96 h after birth (22% for <500 g, 21% for 500–749 g and 22% for 750–999 g). For the two groups with birthweight of 1,000 g or more, the largest number of respondents answered that it reaches 20 mL/kg/day 24–48 h after birth (35% for 1,000–1,249 g and 37% for 1,250–1,499 g), followed by 48–72 h after birth (28% for 1,000–1,249 g and 27% for 1,250–1,499 g).

Question 26: “Which infants, in terms of their gestational age, do you sedate?” (Only for facilities where infants are sedated early after birth.) Eighty‐three respondents (61%) answered that they sedate infants. Among them, the largest number, 28 respondents (33%), answered that they sedate infants born at less than 26 weeks’ gestation, followed by 17 respondents (20%) who answered that they sedate infants born at less than 25 weeks’ gestation and 13 respondents (15%) who answered that they sedate infants born at less than 27 weeks’ gestation. Free description responses included the statement that they sedate all infants with pulmonary hypertension or during intubation.

Question 27: “Until when do you sedate infants?” The largest number of respondents, 78 (more than 90%), answered that they sedate up to 72 h after birth, followed by five respondents who answered that they sedate up to 48 h after birth. Free description responses included statements that the duration is changed according to findings related to pulmonary hypertension, that they sedate infants until there is no risk of intraventricular hemorrhage and that they decide the duration considering the status of cerebral blood flow and ductus arteriosus.

Question 28: “Which amino acid preparations do you use?” One hundred thirty‐three respondents (97%) answered that they use Pleamin‐P® Injection (Fuso Pharmaceutical Industries, Ltd, Osaka, Japan). One respondent did not answer and the answers of three respondents were other than Pleamin‐P®, including AMlZET®B (Terumo Corporation, Tokyo, Japan), AMINOLEBAN® Injection (Otsuka Pharmaceutical Co., Ltd, Tokyo, Japan) and AMIPAREN® Injection (Otsuka Pharmaceutical Co., Ltd, Tokyo, Japan).

Question 29: “Do you use commercially available fluid preparations during the acute phase (within 2 weeks after birth) in VLBW infants?” Ninety‐four respondents (68%) answered that they do, 42 (30%) answered that they do not, and one did not answer. Among the positive answers, Physio®35 Injection (10% glucose added maintenance medium; Otsuka Pharmaceutical Co., Ltd, Tokyo, Japan) was most frequently mentioned as a preparation used and others were SOLDEM® 3A (maintenance medium; Terumo Corporation, Tokyo, Japan) and SOLITAX®‐H (12.5% glucose added hypertonic maintenance medium, AY Pharmaceuticals Co., Ltd, Tokyo, Japan).

## Appendix 3 Additional survey questions

**Table:**

Question 23.	What is the approximate upper limit of acceptable weight loss rate? (by weight group)
	a) Less than 10% b) Less than 15% c) Less than 20% d) Other
Question 24.	Which time point do you refer to for the weight to use for calculating the fluid volume to give, before the weight is restored to the level at birth?
	a) Weight at birth b) Body weight of the day measured every day c) Weight at maximum weight loss d) Other
Question 25.	When does the volume of EN (injection) reach 20 mL/kg/day? (by weight group)
	a) Within 12 h b) 12–24 h after birth c) 24–48 h after birth d) 48–72 h after birth
	e) 72–96 h after birth f) 96–120 h after birth g) 120–144 h after birth h) Other
Question 26.	Which infants, in terms of their gestational age, do you sedate? (only for facilities where infants are sedated early after birth.)
	(free description response)
Question 27.	Until when do you sedate infants?
	(free description response)
Question 28.	Which amino acid preparations do you use?
	a) Pleamin‐P® Injection b) Other → (free description response)
Question 29.	Do you use commercially available fluid preparations during the acute phase (within 2 weeks after birth) in VLBW infants?
	a) Yes → (free description response) b) No

EN, enteral nutrition; VLBW, very low birthweight.
